# *NEAT1* can be a diagnostic biomarker in the breast cancer and gastric cancer patients by targeting XIST, hsa-miR-612, and MTRNR2L8: integrated RNA targetome interaction and experimental expression analysis

**DOI:** 10.1186/s41021-022-00244-3

**Published:** 2022-05-17

**Authors:** Mansoureh Azadeh, Ali Salehzadeh, Kamran Ghaedi, Soheila Talesh Sasani

**Affiliations:** 1 Department of Biology, Rasht Branch, Islamic Azad University, Rasht, Iran; 2 Department of Cell and Molecular Biology and Microbiology, Faculty of Biological Science and Technology, University of Isfahan, Isfahan, Iran; 3 Department of Biology, Faculty of Science, University of Guilan, Rasht, Iran

**Keywords:** Breast cancer, lncRNA, Gene expression, Microarray, Bioinformatics

## Abstract

**Background:**

The most frequent malignancy in women is breast cancer (BC). Gastric cancer (GC) is also the leading cause of cancer-related mortality. Long non-coding RNAs (lncRNAs) are thought to be important neurotic regulators in malignant tumors. In this study, we aimed to evaluate the expression level of *NEAT1* and the interaction of this non-coding RNA with correlated microRNAs, lncRNAs, and mRNAs or protein coding genes, experimentally and bioinformatically.

**Methods:**

For the bioinformatics analyses, we performed RNA-RNA and protein–protein interaction analyses, using ENCORI and STRING. The expression analyses were performed by five tools: Microarray data analysis, TCGA data analysis (RNA-seq, R Studio), GEPIA2, ENCORI, and real-time PCR experiment. qRT-PCR experiment was performed on 50 GC samples and 50 BC samples, compared to adjacent control tissue.

**Results:**

Based on bioinformatics and experimental analyses, lncRNA *NEAT1* have a significant down-regulation in the breast cancer samples with tumor size lower than 2 cm. Also, it has a significant high expression in the gastric cancer patients. Furthermore, *NEAT1* have a significant interaction with XIST, hsa-miR-612 and MTRNR2L8. High expression of *NEAT1* have a correlation with the lower survival rate of breast cancer samples and higher survival rate of gastric cancer patients.

**Conclusion:**

This integrated computational and experimental investigation revealed some new aspects of the lncRNA *NEAT1* as a potential prognostic biomarker for the breast cancer and gastric cancer samples. Further investigations about NEA1 and correlated mRNAs, lncRNAs, and microRNAs – specially the mentioned RNAs in this study – can lead the researchers to more clear information about the role of *NEAT1* in the breast cancer and gastric cancer.

## Introduction

Breast cancer (BC) is the most common cancer type in women. Long non-coding RNAs (lncRNAs) are considered crucial gene expression controllers in malignant growths [1]. Gastric cancer (GC) can also be considered the most significant cause of cancer-related death [2]. Among the various known types of lncRNAs, Nuclear Enriched Abundant Transcript 1 (NEAT1), with five recognized splice structures, is situated on chromosome 11. Based on the Gene cards, this gene delivers a long non-coding RNA (lncRNA) deciphered from the numerous endocrine neoplasia locus. This lncRNA is held in the core, where it frames the center primary part of the paraspeckle sub-organelles. It might go about as a transcriptional controller for a very long time, incorporating a few qualities associated with disease movement. NEAT1 can enable miRNA binding and RISC complex binding. This lncRNA has an important role in the positive inflammatory response regulation, negative regulation of gene silencing by miRNA, and positive regulation of synoviocyte proliferation [3].

Mutation in the promoter region of NEAT1 can lead the normal breast and renal cells to the carcinoma [4, 5]. In hepatocellular carcinoma cells, treatment with 5-AZA enhanced NEAT1 expression, demonstrating that DNA methylation is a significant determinant of NEAT1 expression [6]. Based on previous studies, NEAT1 controls the initiation and progression of cancer by three molecular mechanisms: (i) influencing the expression level of downstream factors of EZH2, by acting as a scaffold RNA molecule for EZH2, (ii) playing crucial role as a miRNA sponge to disrupt the connections of several tumor suppressor miRNAs with their target mRNAs, and (iii) suppressing the expression of miR-129 (promotion of DNA methylation in the promoter region of miR-129) [7].

Recent studies revealed that NEAT1 has a significant role in developing different cancer types. For example, Xuesong Wang et al. at 2020 found that lncRNA NEAT1 had a significantly high expression in colorectal cancer (CRC) tissues and cells. Also, they demonstrated that the knockdown of NEAT1 can promote the invasion and apoptosis of CRC cells. They also find a novel microRNA (miR-150-5P) that NEAT1 can sponge [8]. In May 2020, Gao M et al. demonstrated that NEAT1 could promote the progression of GC by sponging miR-356a-3p and regulating ABCC4 [9]. NEAT1 expression may be controlled by signal transducer and activator of transcription 3, and changed NEAT1 expression epigenetically influences downstream gene transcription during herpes simplex virus-1 infection and Alzheimer's disease, implying that NEAT1 functions as a stress sensor and effector. The chemicals and regulatory patterns that control NEAT1 gene expression, as well as the molecular mechanism by which NEAT1 regulates the expression of its target genes, are summarized and discussed in this study, bringing new insights into NEAT1's essential function in gene regulation [10]. This molecule also can regulate the regulate the liver fibrosis in the alcoholic steatohepatitis [11]. By modulating the TLR2/NF-B signaling pathway, the LncRNA NEAT1 reduces sepsis-induced myocardial damage [12]. The repressor complex FOXN3-NEAT1-SIN3A promotes the development of hormonally sensitive breast cancer [13]. Both depletion of mitochondrial proteins and treatment of mitochondrial stressors result in abnormal NEAT1 expression as well as changes in the shape and quantity of paraspeckles via ATF2. The retention of mRNAs of nuclear-encoded mitochondrial proteins (mito-mRNAs) in paraspeckles is improved as a result of these alterations. NEAT1 depletion, on the other hand, has a significant impact on mitochondrial dynamics and function through affecting mito-mRNA sequestration in paraspeckles [14].

NEAT1 is a nuclear architectural long non-coding RNA with a high abundance. NEAT1-1 and NEAT1-2 are two overlapping NEAT1 isoforms, with the latter serving as a scaffold for the construction of paraspeckles, a type of nuclear ribonucleoprotein body [15]. It was recently discovered that NEAT1-2 expression, but not NEAT1-1, predicts progression-free survival in ovarian cancer patients receiving platinum-based treatment [16]. Erik Knutsen et al. showed that NEAT1-2 expression level is significantly associated with the breast cancer tumor grade and HER2 positive breast cancer samples. Also, during lactation, NEAT1-2 expression is increased in human breast tissue [17]. Previous studies revealed that NEAT1-1 is expressed in a variety of cell types in the adult mouse tissue. however, the epithelial layers of digestive tissues are where NEAT1-2 is mostly expressed [7].

Mentioned information about the role of NEAT1 reveals that this non-coding RNA is a crucial molecule in regulating different biological processes and pathological statuses. Unwanted changes in the expression of this lncRNA can lead the cells into different diseases, including breast cancer and gastric cancer. In this study, we aimed to evaluate the differences in the expression level of NEAT1 in the high-throughput breast cancer and gastric cancer datasets and the human GC and BC samples of the Isfahan population. Also, we find an RNA regulatory interaction network that can regulate NEAT1 expression level and evaluate the expression of the most significant hub RNAs in this network, directly or indirectly.

## Materials and methods

### Microarray analysis

Microarray data analysis was performed on the two gastric and breast cancer datasets. GSE54129 was analyzed for finding the differentially expressed genes (DEGs) in the gastric cancer microarray samples. Twenty-one control samples and 111 GC samples in this dataset were analyzed. Also, this study analyzed GSE10810 [18] with 27 control samples and 31 case samples to find the DEGs in the BC. These datasets are provided by GPL570 ([HG-U133_Plus_2] Affymetrix Human Genome U133 Plus 2.0 Array). The raw data were downloaded from GEO online database (https://www.ncbi.nlm.nih.gov/geo/) and moved to the R Studio environment and normalized by the affy package (Read.Affy command in R) [19]. Statistical analysis of the microarray dataset was performed by limma package [20]. Affy and limma packages were downloaded from Bioconductor online database (https://www.bioconductor.org/). The significance level of microarray data analysis was considered as 0.05 (adjusted p value). The plots of microarray data analysis were created by the ggplot2 and pheatmap packages, downloaded from Bioconductor (Bioconductor.org). In this microarray analysis, the expression of 54,675 mRNA and lncRNA transcripts were analyzed. After normalization (quantiles normalization method) of raw data, transforming the expression data into logarithmic scale, and deleting the transcripts with no expression in the dataset, the difference in the expression level of all RNAs was calculated. The RNAs with logFC > 3 were selected as the up-regulated RNAs and the logFC < -3 was selected as the threshold of low expression.

### Bioinformatics analyses

The RNA interaction analyses (miRNA-lncRNA, mRNA-lncRNA, and lncRNA-lncRNA) were performed by ENCORI online database [21] (https://starbase.sysu.edu.cn/). Expression and survival analyses was performed by GEPIA2 [22] (http://gepia2.cancer-pku.cn/) and ENCORI online software. RNA interactions were visualized by Cytoscape (3.8.2) software [23]. Protein–protein interaction analysis was performed by the STRING online database [24].

### TCGA RNAseq data analysis

The next generation sequencing dataset (RNAseq) from BRCA (Breast cancer) project of The Cancer Genome Atlas (TCGA) database (https://portal.gdc.cancer.gov) with 1109 BC samples (1102 primary solid tumor and 7 metastatic tumor samples) and 113 solid tissue normal samples were downloaded. TCGAbiolinks, edgeR, and limma (Voom method) packages were used for obtaining the datasets, normalization, batch effect removal, statistical analyses, and differential expression analysis. Normalization of raw data was performed by the TMM (trimmed mean of M-values) method.

### Clinical characteristics of tissue samples

The Ethics Committee of Al-Zahra Hospital, Isfahan University of Medical Science, approved all procedures for the research in this study that involved human samples, and all patients signed written consent forms. Breast cancer and surrounding normal breast tissue samples from 50 individuals with breast cancer were analyzed in a case–control study. Also, the same expression analysis was performed on 50 gastric cancer samples compared to 50 adjacent normal samples. None of the patients had previously had radiation or chemotherapy. Tissue biopsies were rinsed in distilled water before being submerged in RNA later solution (Invitrogen, USA) and quickly preserved in liquid nitrogen for pathologist evaluation. The clinicopathological characteristics of breast cancer and gastric cancer patients are provided in Table 1 and Table 2.

**Table 1:** Clinicopathological variables of BC patients

Characteristic	Status	Number of patients
Stage	I	8 (16%)
	II	15 (30%)
	III	12 (24%)
	IV	9 (18%)
	Unknown	6 (12%)
Age	< 45	20 (40%)
	> 45	24 (48%)
	Unknown	6 (12%)
Lymph node metastasis	Yes	17 (34%)
	No	26 (52%)
	Unknown	7 (14%)
Tumor size (TS)	< 2 cm	15 (30%)
	5 > TS > 2	5 (10%)
	> 5 cm	29 (58%)
	Unknown	1 (2%)
Menopausal status	Yes	12 (24%)
	No	15 (30%)
	Unknown	23 (46%)
ER receptor	Positive	14 (28%)
	Negative	13 (26%)
	Unknown	23 (46%)
PR receptor	Positive	19 (38%)
	Negative	21 (42%)
	Unknown	10 (20%)
HER2/neu receptor	Positive	12 (24%)
	Negative	21 (42%)
	Unknown	17 (34%)

**Table 2:** Clinicopathological variables of GC patients

Characteristic	Status	Number of patients (%)
Blood Group	A +	9 (18%)
	B +	7 (14%)
	B-	11 (22%)
	O +	5 (10%)
	O-	3 (6%)
	AB	12 (24%)
	Unknown	4 (8%)
Smoking	Yes	14 (28%)
	No	36 (72%)
	Unknown	0
H. Pylori	Positive	12 (24%)
	Negative	21 (42%)
	Unknown	17 (34%)
Tumor Stage	I	12 (24%)
	II	11 (22%)
	III	8 (16%)
	IV	13 (26%)
	Unknown	6 (12%)
Metastasis	Positive	19 (38%)
	Negative	21 (42%)
	Unknown	10 (20%)
Tumor Size	> 6 cm	17 (34%)
	< 6 cm	31 (62%)
	Unknown	2 (4%)
Tumor location	Greater curvature	17 (34%)
	Lesser curvature	12 (24%)
	Cardia	10 (20%)
	Antrum	11 (22%)
	Unknown	0
Histological type	Mucinous adenocarcinoma	11 (22%)
	Intestinal	12 (24%)
	Signet ring cell	16 (32%)
	Unknown	6 (12%)

### Real-time PCR

The total RNA content of breast cancer tissue samples and normal breast tissue equivalents from the same individuals was acquired and extracted according to the manufacturer's procedure using an RNA extraction kit (GeneAll, Seoul, Korea). According to the manufacturer's procedure, the first-strand cDNA synthesis kit (Thermo Fisher Scientific, Waltham, MA, USA) was used to make cDNA. The cDNA products were stored at -20 ◦C for the expression analysis of NEAT1 and GAPDH as the reference gene. Using oligo 7 software, the specific primers were designed for the NEAT1 and GAPDH (Table 3). The qRT-PCR experiment was performed using Magnetic Induction Cycler (MIC) (Bio molecular Systems, Australia).

**Table 3:** Primer sequence table

Gene	Forward / Reverse	Primer sequence
*NEAT1*	Forward	5’CTTCTTCCCTTTAACTTATCCATTCAC3’
	Reverse	5’CTCTTCCTCCACCATTACCAACAATAC3’
*GAPDH*	Forward	5’ACAGGGTGGTGGACCTCAT3’
	Reverse	5’AGGGGTCTACATGGCAACTG3’

### Statistical analysis

GraphPad Prism software performed statistical analysis of real-time PCR data and the related graphs (version 8). qRT-PCR data were analyzed using the 2− ΔΔCT method to compare expression levels between the tumor and control samples. The Shapiro-Wilk test was performed on the expression data to evaluate the normality of data. Paired t-test was performed on the -ddCt data to compare expression levels in tumor and control samples. DEG analysis of microarray and the TCGA dataset was performed by R Studio (4.1.0). The GraphPad prism performed the ROC analysis for the real-time PCR datasets based on sensitivity and specificity. P-value less than 0.05 was considered as the significance level of this study. In the ROC analysis, AUC between 0.7 – 0.8 is a fair AUC value, AUC between 0.8-0.9 is a good AUC value (indicating a good biomarker), and AUC between 0.9-1 revealed an excellent biomarker.

## Results

### *NEAT1* had a significantly high expression in breast cancer and low expression in the gastric cancer tissue

**Fig. 1:**
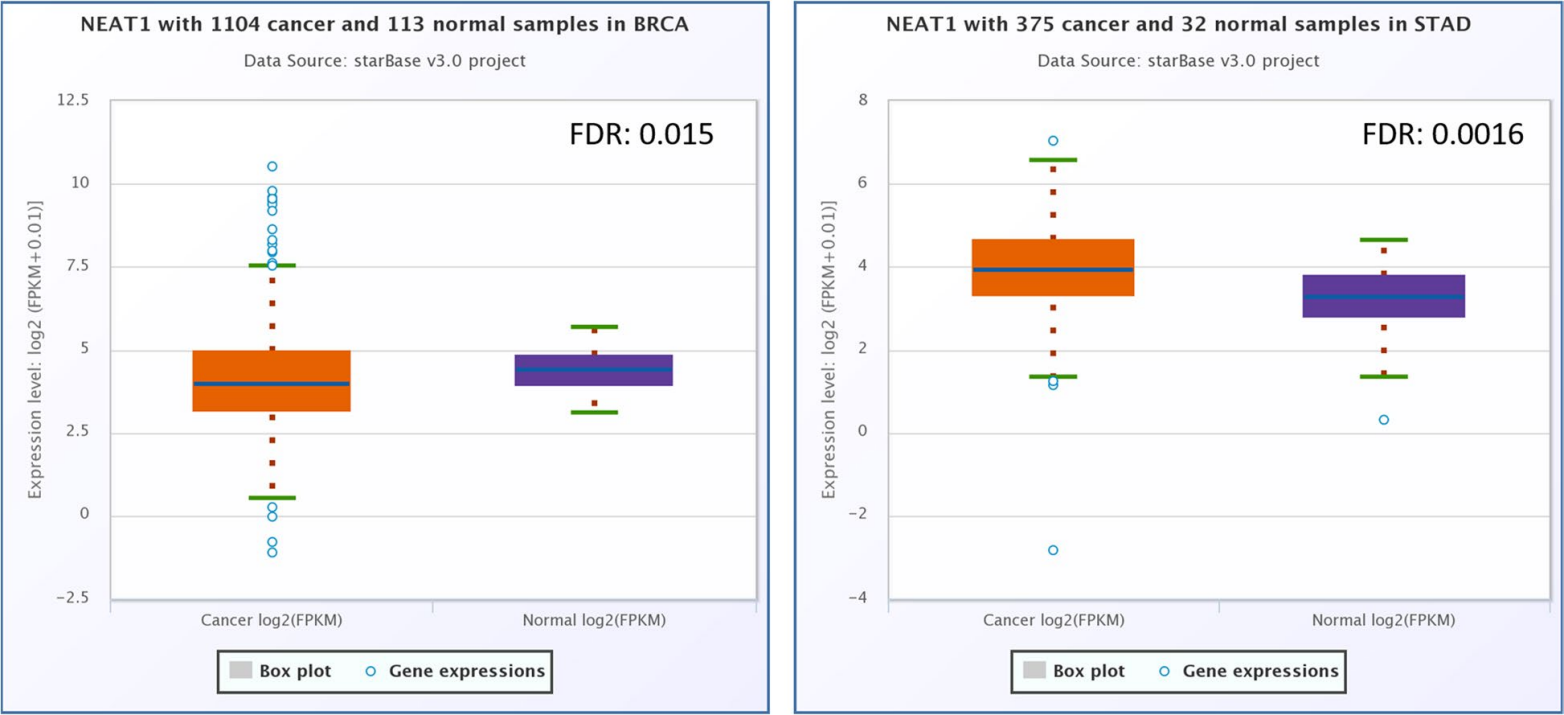
Expression analysis of *NEAT1* in the gastric cancer samples and breast cancer samples, compared to control samples, based on the ENCORI online software. This analysis showed that *NEAT1* had a significant up-regulation in the GC samples and a significantly low expression in the BC samples

**Fig. 2:**
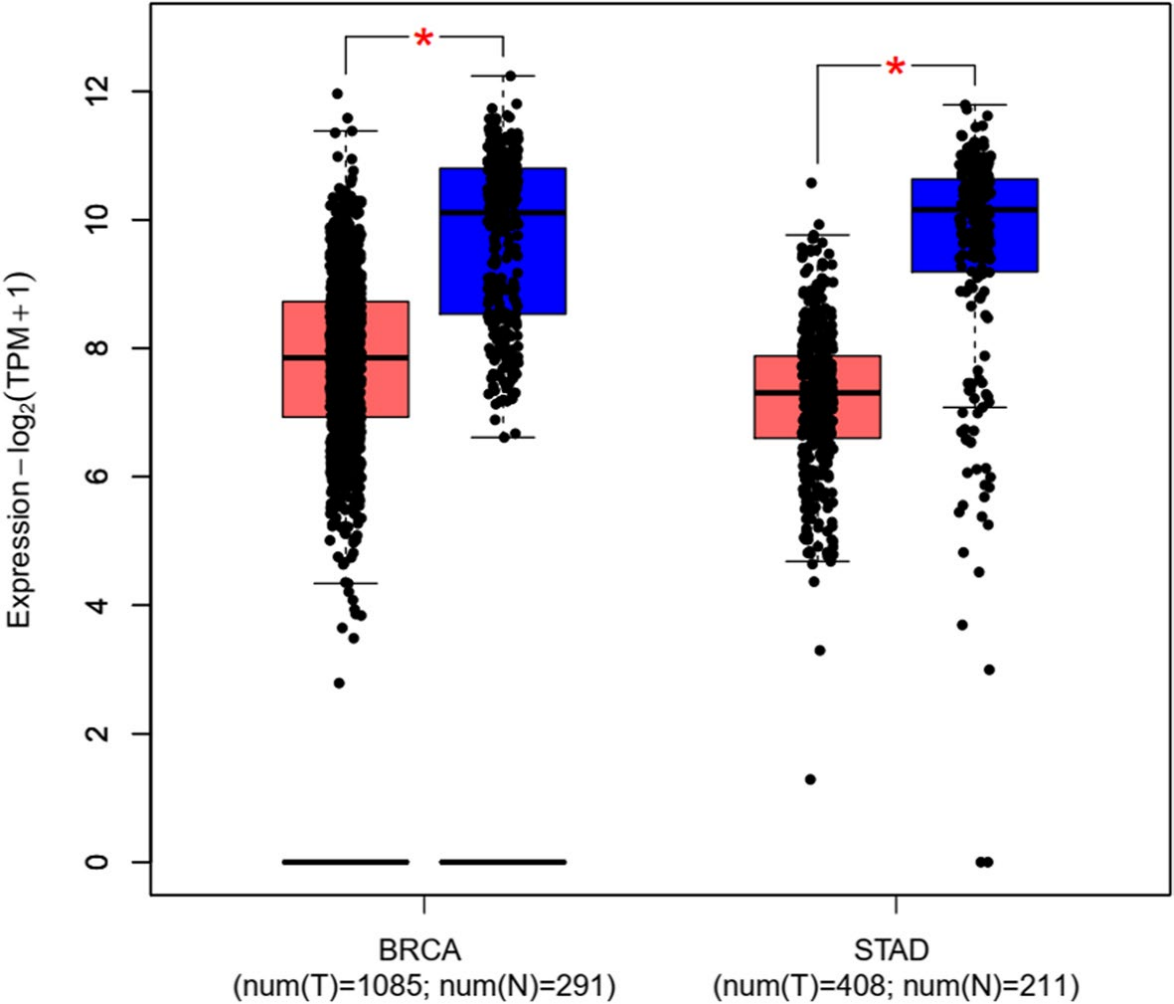
Relative expression analysis of GEPIA2 online software, based on the TCGA RNA-seq datasets. According to the GEPIA2 data analysis, *NEAT1* had a significantly low expression in the GC and BC samples compared to control samples

**Fig. 3:**
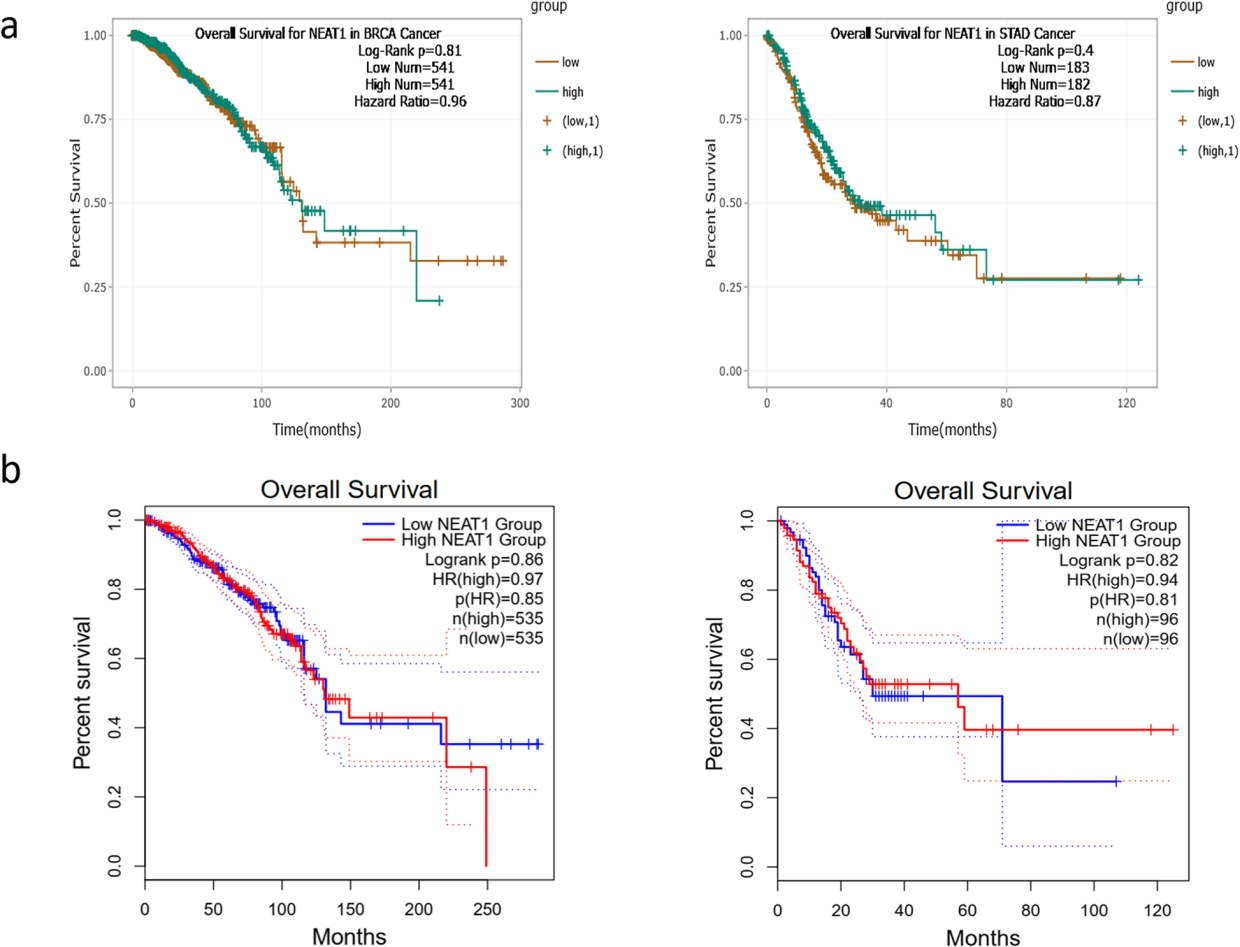
Survival analysis of ENCORI and GEPIA2 online databases, based on the expression level of *NEAT1* in the BC and GC patients. **a**) Survival analysis by ENCORI online database revealed a not significant negative correlation between the expression level of *NEAT1* in the BC patients and the survival rate of BC samples. **b**) GEPIA2 online survival analysis revealed that the high *NEAT1* expression is correlated with the lower survival rate of BC samples and higher survival rate of GC patients, not significantly

Based on the ENCORI (Fig. 1) and data analysis, NEAT1 has a significant down-regulation in breast cancer (FC: 0.73, FDR: 0.015) and gastric cancer (FC: 1.81, FDR: 0.0016). GEPIA2 (Fig. 2) online data analysis revealed that NEAT1 had a significant downregulation in the breast cancer and gastric cancer samples. Survival analysis by ENCORI and GEPIA2 revealed that high expression of the NEAT1 has a non-significant correlation with the low survival rate of BC patients. Also, the survival analysis revealed that the low-expression of NEAT1 has a not-significant relation with the low survival rate of GC patients (Fig. 3).

### Real-time PCR data analysis of *NEAT1* in the BC and GC samples, compared to control (Isfahan population)

**Fig. 4:**
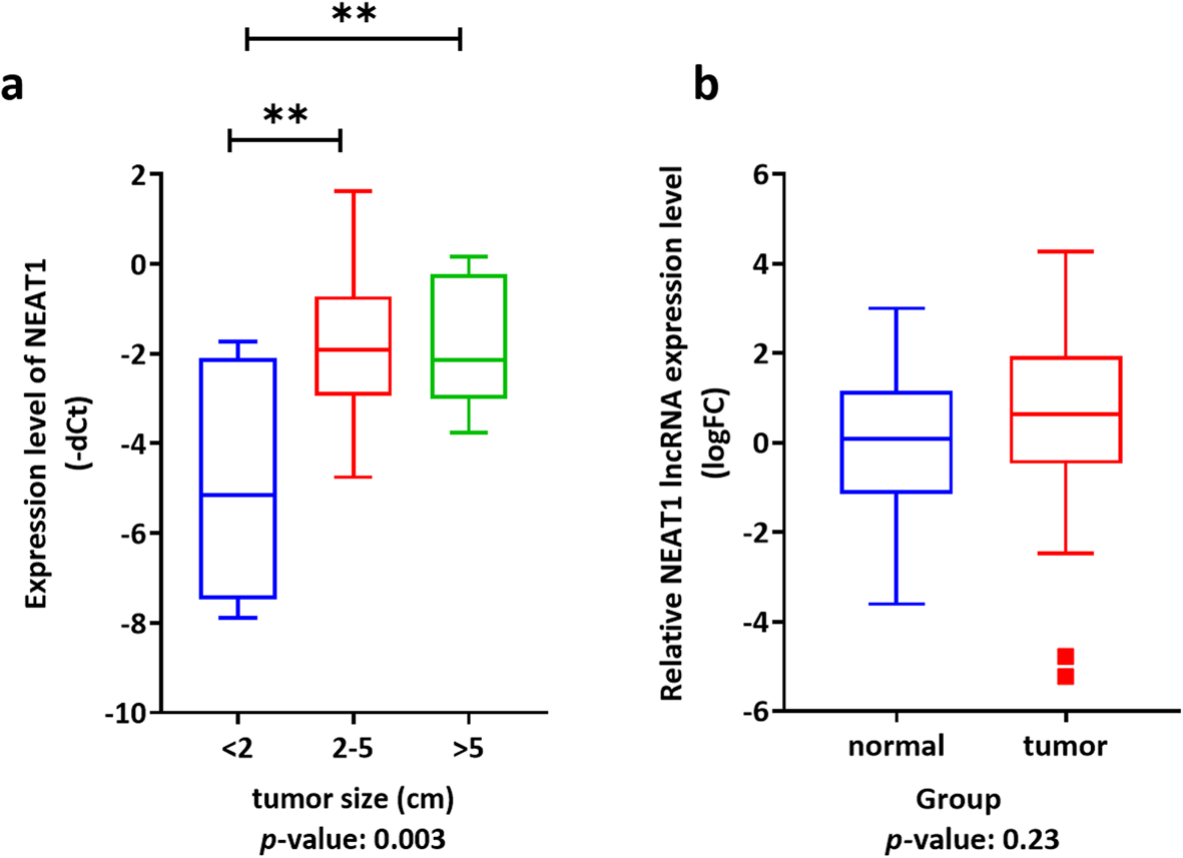
Relative expression analysis of *NEAT1* in the breast cancer samples revealed that *NEAT1* expression levels have a significant down-regulation in the breast cancer samples with tumor size lower than 2 cm, compared to the samples with bigger tumor size

Real-time PCR data analysis of lncRNA expression level revealed that the expression of this lncRNA had no significant difference in the breast cancer cohort compared to the control. However, there was a significant dysregulation in expression level in the different clinicopathological situations. There was a significant up-regulation in expression level in the samples with the size of tumor bigger than 2 cm, compared to the samples smaller than 2 cm (Fig. 4).

**Fig. 5:**
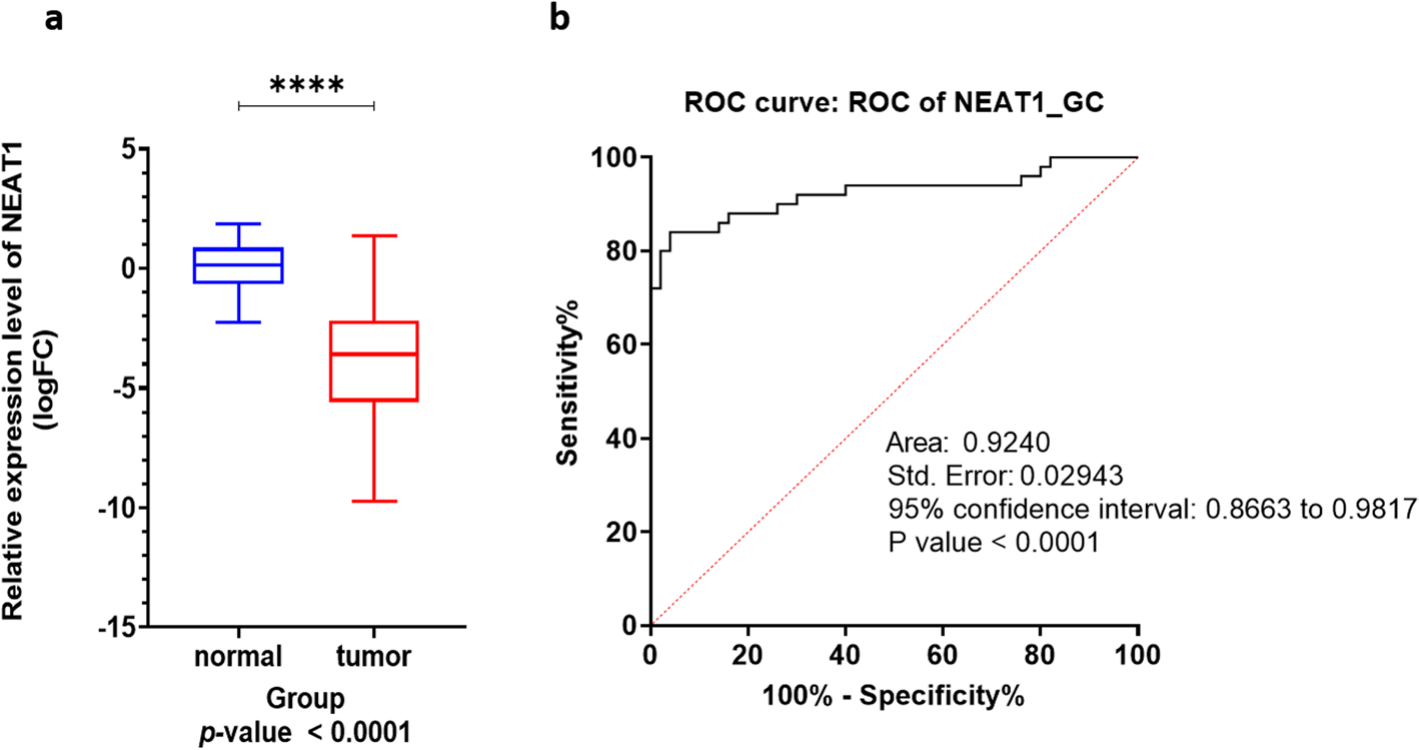
Real-time PCR data analysis of the *NEAT1* expression in the GC samples, compared to control. **a** Relative expression analysis of *NEAT1* revealed that this non-coding RNA had a significantly low expression in the GC patients. **b** ROC analysis revealed that *NEAT1* could be an excellent prognostic biomarker for the Isfahan GC population

Real-time PCR data analysis revealed that NEAT1 has a significant down-regulation in the Isfahan human gastric cancer samples, compared to control (logFC: -3.775, *p*-value < 0.0001, Fig. 5a). ROC analysis revealed that NEAT1 can be an excellent prognostic biomarker for the Isfahan gastric cancer patient and can be a novel factor for distinguishing the tumor samples from control samples (AUC: 0.924, *p*-value < 0.0001, Fig. 5b).

For validation of mentioned results about the expression of NEAT1, the TCGA data analysis was performed on the breast cancer RNA-seq dataset. TCGA data analysis revealed that the expression level of lncRNA NEAT1 has a significant up-regulation in high-throughput Breast cancer samples compared to control samples (logFC: 8.906262, adj. *P*. Value < 0.0001).

### Integrated RNA interaction analysis

**Fig. 6:**
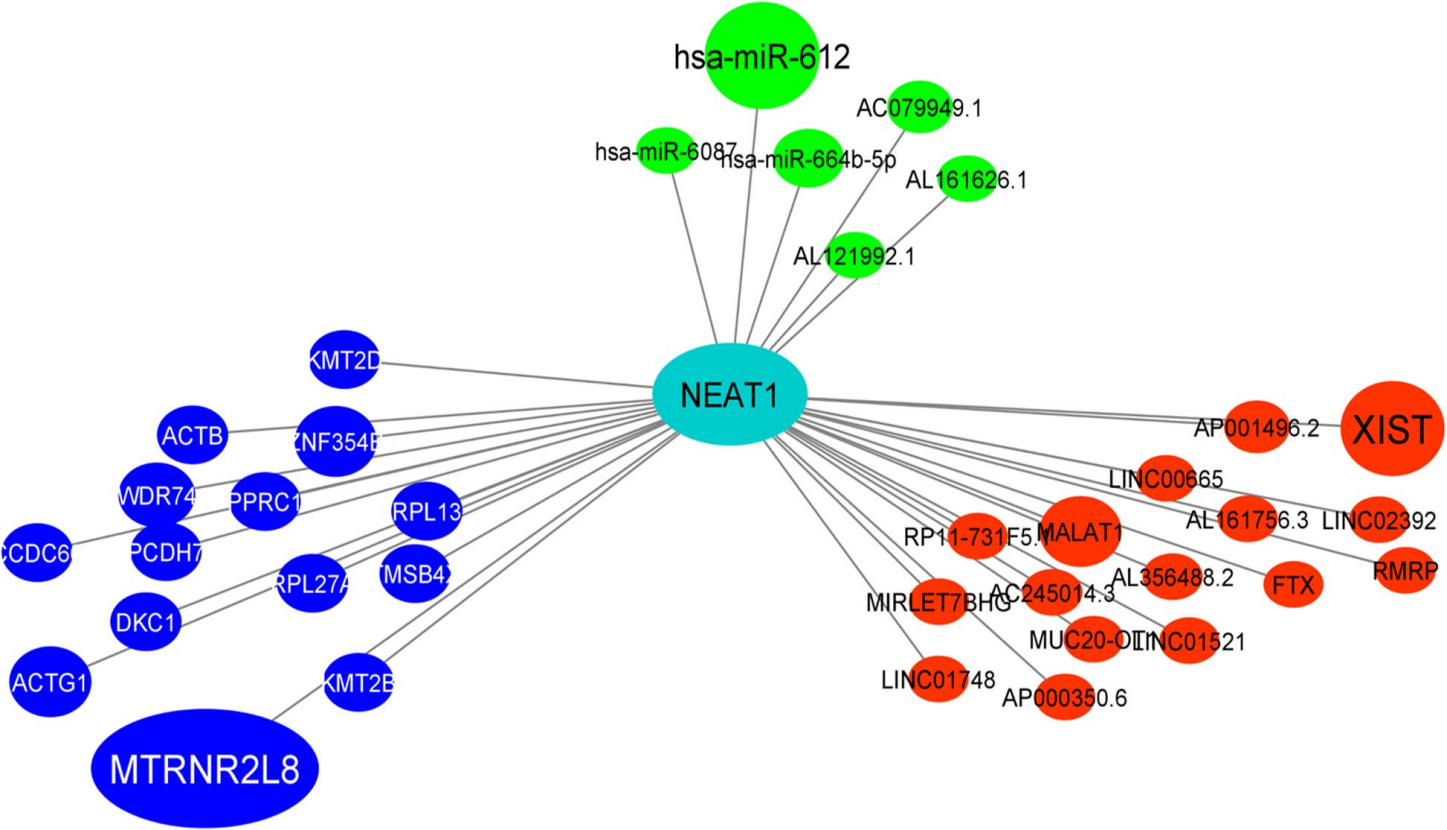
The RNA-interaction network of *NEAT1*, based on the ENCORI online data analysis. The red color indicates the lncRNAs, blue indicates mRNAs, and green indicates microRNAs that have RNA interactions with *NEAT1*. The size of the nodes is positively correlated with the interaction number

**Fig. 7:**
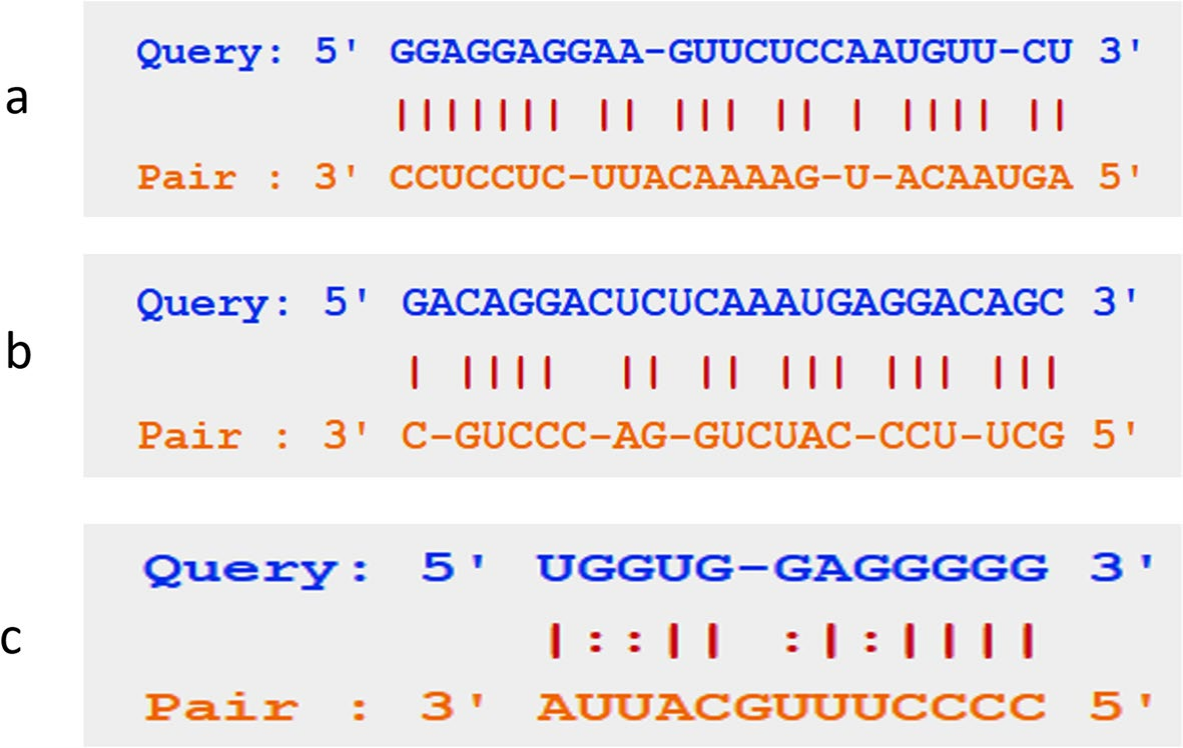
RNA interaction of *NEAT1* with *MTRNR2L8* **a**, hsa-miR-612 **b**, and XIST **c**

For finding the RNA and protein interaction network surrounding the NEAT1, ENCORI online database was used. Based on the interaction number of RNAs with NEAT1, the hub mRNAs, miRNAs, and lncRNAs that have significant interactions with NEAT1 were detected (Figs. 6 and 7). Based on this interaction analysis, NEAT1 had a significant RNA interaction with MTRNR2L8 protein-coding RNA (number of interactions: 13, free energy: -25.4, Align score: 30). NEAT1 had a significant RNA interaction with the lncRNA XIST (number of interactions: 9, free energy: -19.8, Align score: 19.5). Also, NEAT1 has significant interaction with hsa-miR-612 (number of interactions: 11, free energy: -18.3, Align score: 26). All of the RNAs in the interaction network are listed in Table 4.

**Table 4:** RNA interaction analysis of *NEAT1*

Gene Name	Pair Gene Name	Pair Gene Type	n	Free Energy	Score (Smith-Waterman)
*NEAT1*	MTRNR2L8	protein_coding	13	-25.4	30
*NEAT1*	ACTG1	protein_coding	5	-32.2	21.5
*NEAT1*	ZNF354B	protein_coding	5	-12.9	19
*NEAT1*	WDR74	protein_coding	4	-21.5	24
*NEAT1*	TMSB4X	protein_coding	3	-35.3	31
*NEAT1*	ACTB	protein_coding	3	-35.1	23.5
*NEAT1*	RPL13	protein_coding	3	-26.6	20.5
*NEAT1*	RPL27A	protein_coding	3	-24.4	18
*NEAT1*	DKC1	protein_coding	3	-22.8	19
*NEAT1*	PPRC1	protein_coding	3	-19.8	15.5
*NEAT1*	PCDH7	protein_coding	3	-15.5	24
*NEAT1*	KMT2D	protein_coding	3	-14.5	16
*NEAT1*	KMT2B	protein_coding	3	-14.1	13.5
*NEAT1*	CCDC66	protein_coding	3	-11.5	11.5
*NEAT1*	XIST	lincRNA	9	-19.8	19.5
*NEAT1*	MALAT1	lincRNA	5	-24.9	20.5
*nNEAT1*	AP001496.2	lincRNA	2	-24.8	17
*NEAT1*	MIRLET7BHG	lincRNA	1	-14	14
*NEAT1*	LINC01521	lincRNA	1	-10.8	14
*NEAT1*	FTX	lincRNA	1	-11	17.5
*NEAT1*	LINC00665	lincRNA	1	-11.1	14
*NEAT1*	LINC01748	lincRNA	1	-8.1	19.5
*NEAT1*	MUC20-OT1	lincRNA	1	-11.6	16
*NEAT1*	RP11-731F5.1	lincRNA	1	-34.9	28
*NEAT1*	LINC02392	lincRNA	1	-22.4	28
*NEAT1*	AL356488.2	lincRNA	1	-11.2	13
*NEAT1*	AP000350.6	lincRNA	1	-9.2	10
*NEAT1*	AC245014.3	lincRNA	1	-12.7	16
*NEAT1*	AL161756.3	lincRNA	1	-22.2	12.5
*NEAT1*	RMRP	lincRNA	1	-9.7	17
*NEAT1*	hsa-miR-612	miRNA	11	-18.3	26
*NEAT1*	hsa-miR-664b-5p	miRNA	3	-22.8	19
*NEAT1*	AC079949.1	miRNA	2	-24.8	13
*NEAT1*	AL161626.1	miRNA	1	-6.5	10
*NEAT1*	AL121992.1	miRNA	1	-16.5	17
*NEAT1*	hsa-miR-6087	miRNA	1	-29.4	23

### Expression analysis of MTRNR2L8, hsa-miR-612, and XIST in the GC and BC samples

**Fig. 8:**
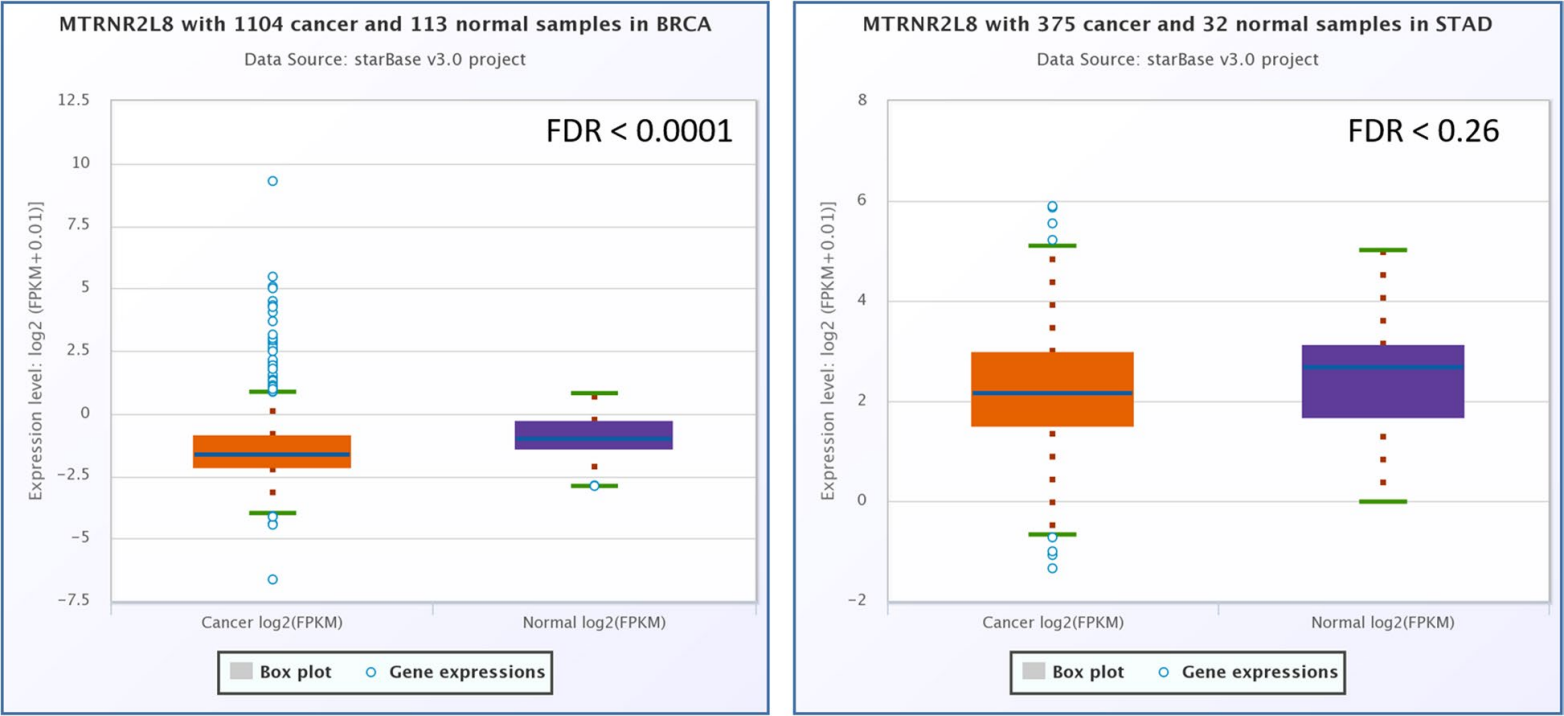
ENCORI elative expression analysis of *MTRNR2L8* in the BC and GC samples

**Fig. 9:**
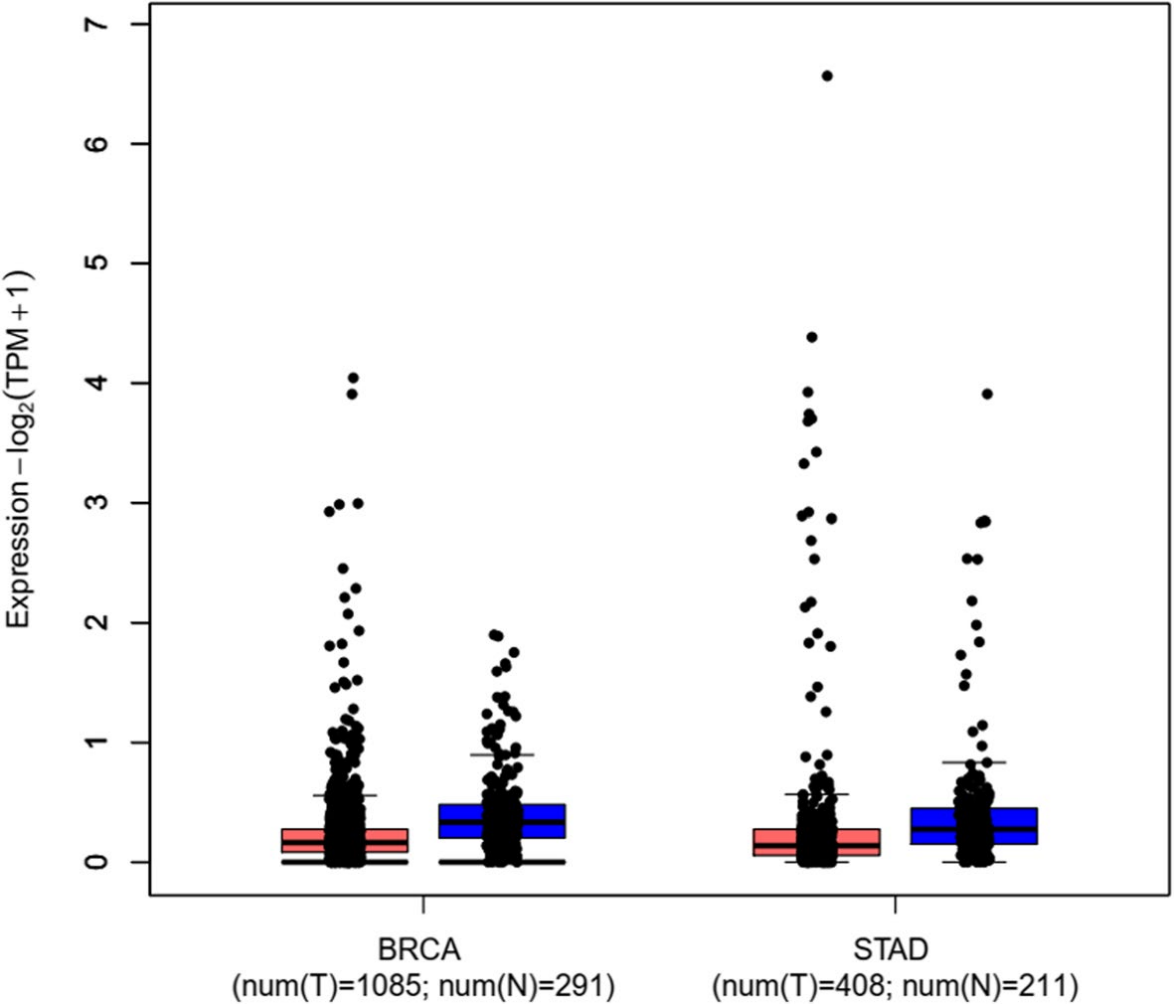
GEPIA2 relative expression analysis of *MTRNR2L8* in the BC and GC samples

**Fig. 10:**
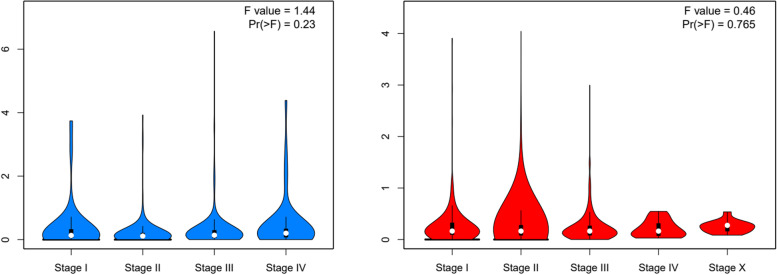
GEPIA2 expression analysis of MTRNR2L8 revealed that there is no significant difference between the expression levels of this gene in the different stages of breast cancer and gastric cancer samples

**Fig. 11:**
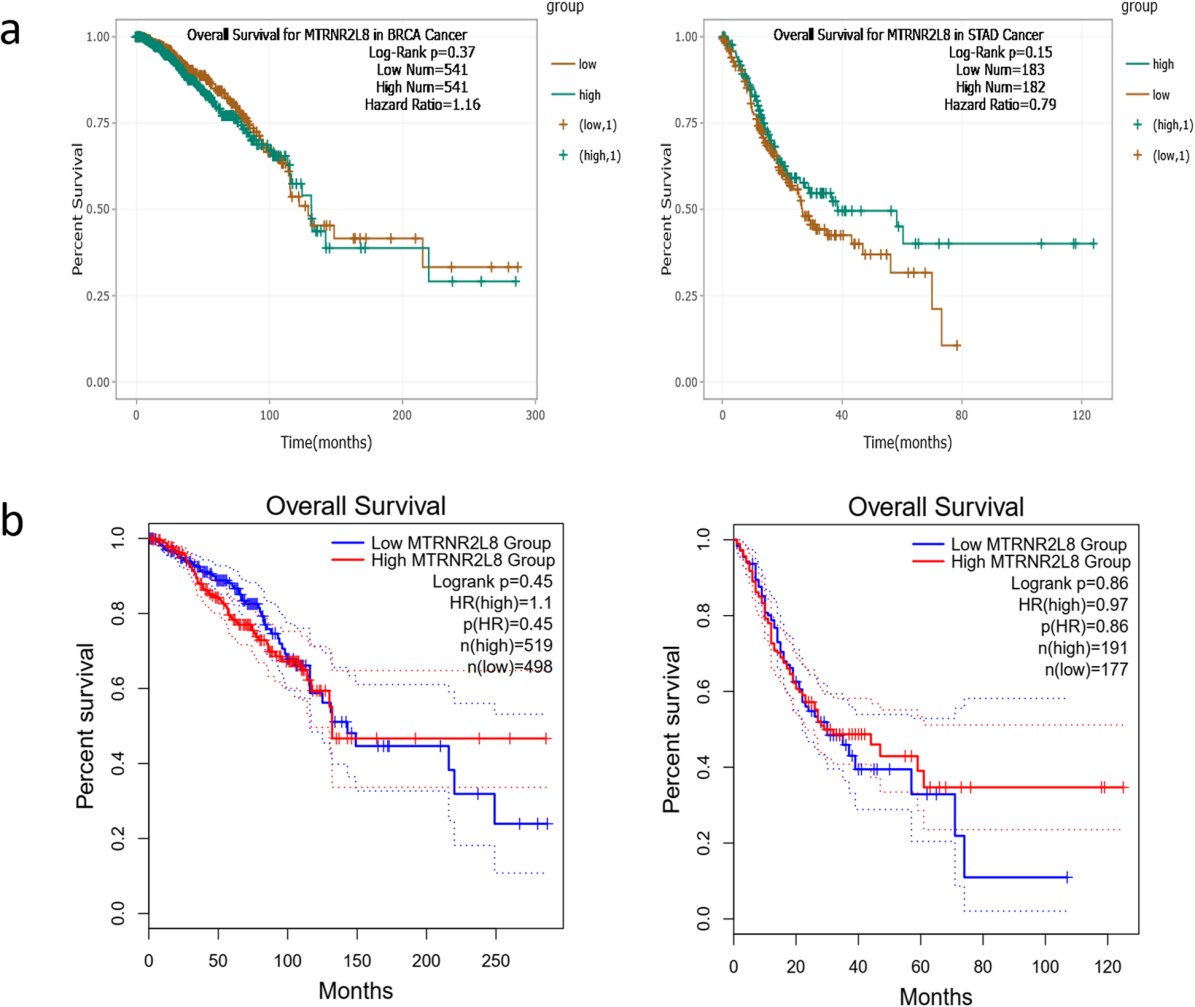
Survival analysis of *MTRNR2L8* in the BC and GC samples, based on the ENCORI **a** and GEPIA2 **b** online databases

After RNA interaction analysis, it is demonstrated that MTRNR2L8, hsa-miR-612, and XIST are three coding and non-coding RNAs that could regulate the expression and activity of NEAT1. For understanding the possible role of mentioned coding and non-coding RNAs in the BC and GC patients, the relative expression analysis of the mentioned RNAs was performed by ENCORI, GEPIA2, and microarray data analyses. Based on the ENCORI data analysis, MTRNR2L8 had a significant low expression in the breast cancer samples (FC: 0.66, FDR < 0.0001) and a not significant low-expression in the GC samples (FC: 0.83, FDR: 0.26, Fig. 8). GEPIA2 online expression analysis revealed no significant dysregulation in the BC and GC samples (Fig. 9). Also, GEPIA2 expression analysis revealed that there is no significant correlation between the stages of breast and gastric cancer and the expression level of MTRNR2L8 (Fig. 10). Survival analysis of ENCORI and GEPIA2 databases revealed a not significant correlation between the low-expression of MTRNR2L8 and the low survival rate of BC and GC patients (Fig. 11). Figure 11 presented a protein–protein interaction for MTRNR2L8.

**Fig. 12:**
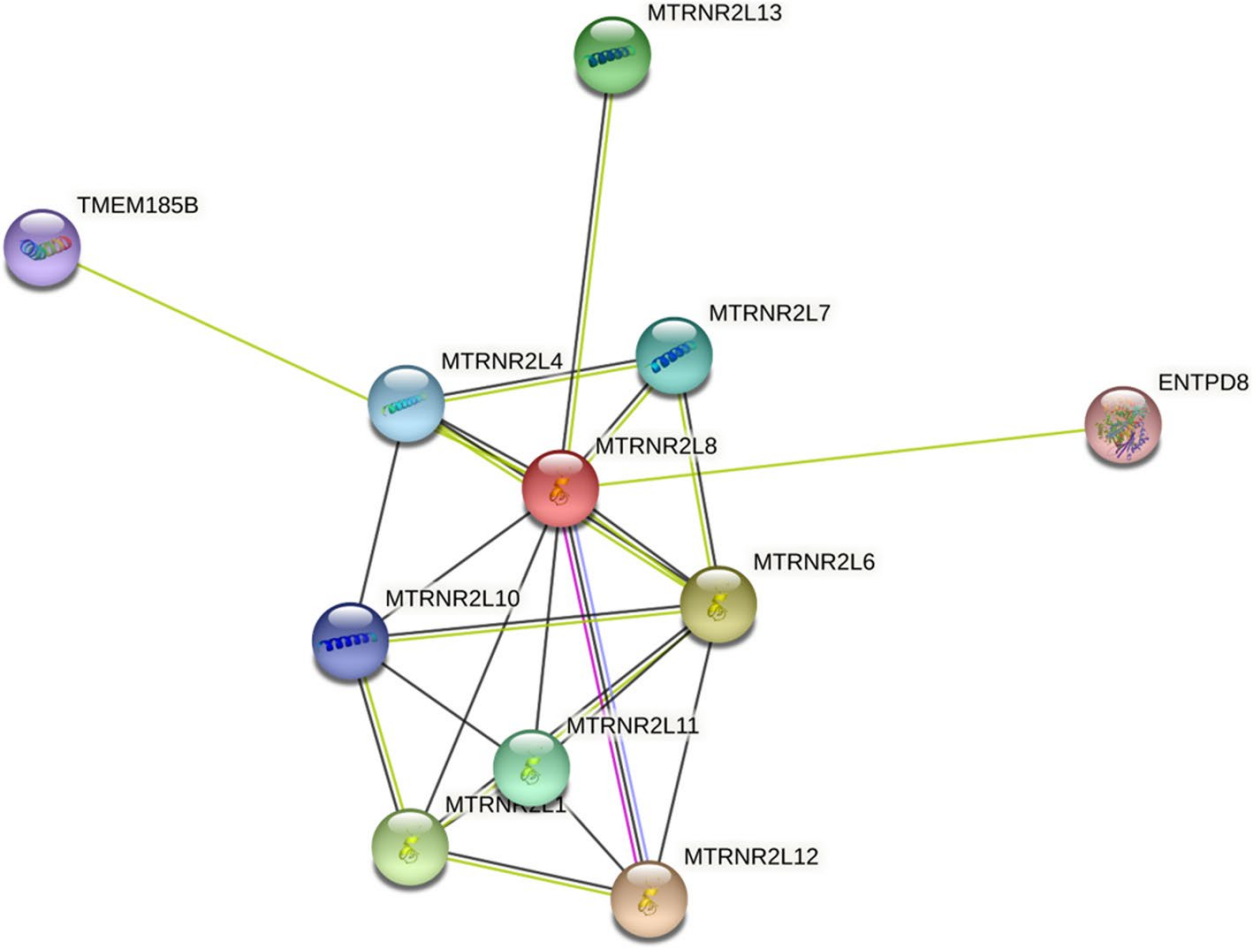
The protein–protein interaction analysis of MTRNR2L8, based on the STRNIG online database

**Fig. 13:**
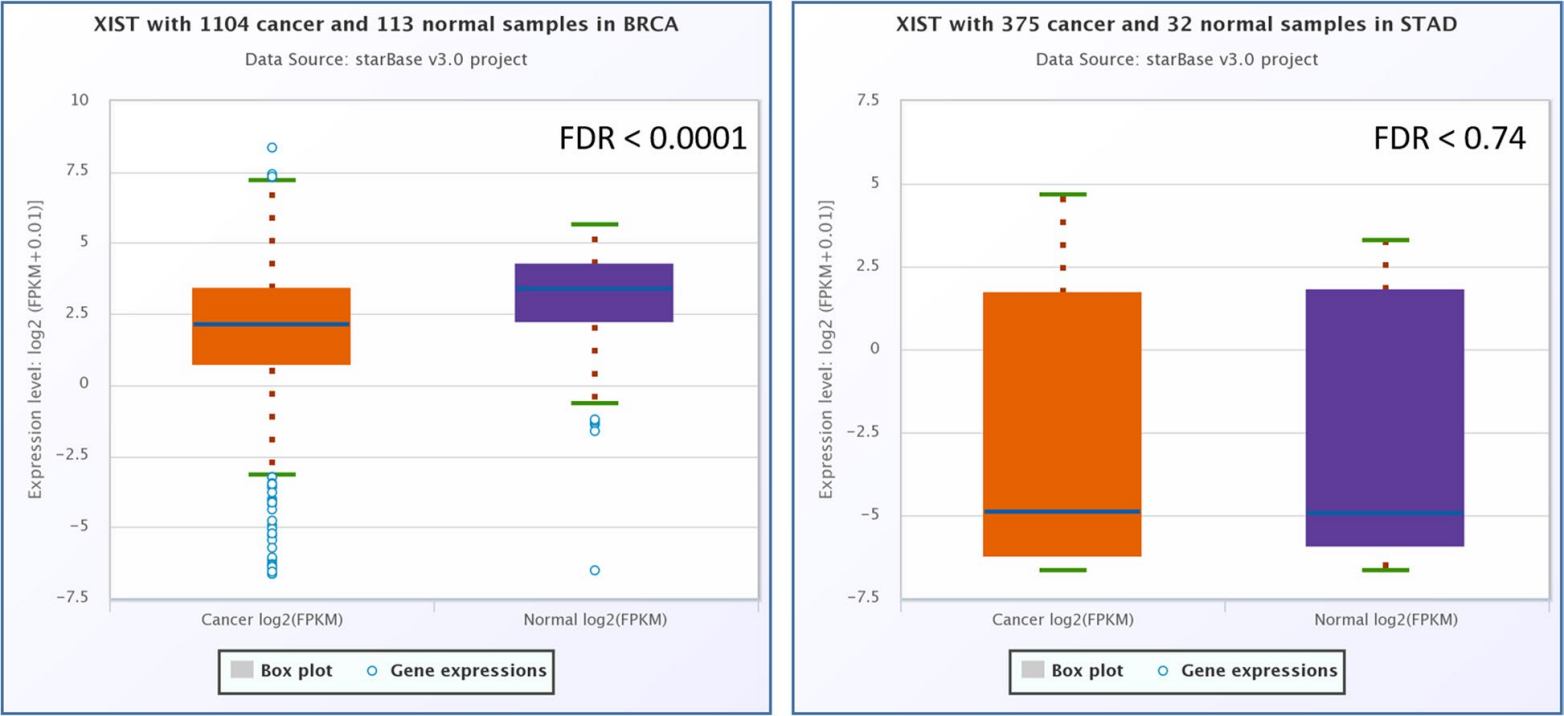
The low-expression of lncRNA XIST in the BC samples, based on the ENCORI relative expression analysis

**Fig. 14:**
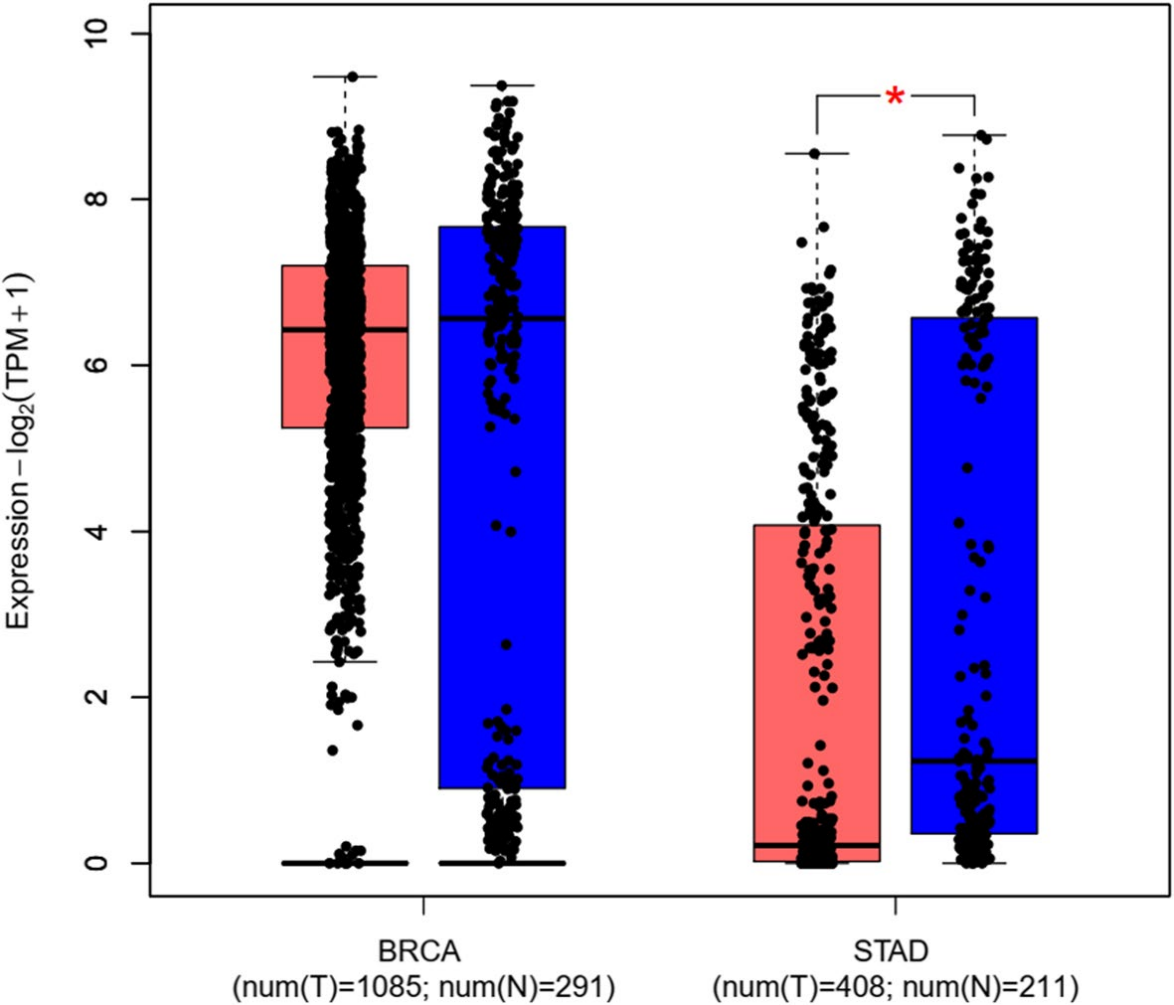
The significant low-expression of lncRNA XIST in the GC samples, based on the GEPIA2 relative expression analysis

The ENCORI relative expression analysis of lncRNA XIST (Fig. 12) revealed that XIST has a significantly low expression in the BC samples (FC: 0.62. FDR < 0.0001) and has a not significant up-regulation in the GC samples (FC: 1.44, FDR: 0.74). GEPIA2 online expression analysis revealed that XIST has a significantly low expression in the GC samples (Fig. 13). Survival analysis revealed no significant correlation between the expression pattern of lncRNA XIST and the survival rate of the BC and GC patients (Fig. 14).

### Microarray data analysis

**Fig. 15:**
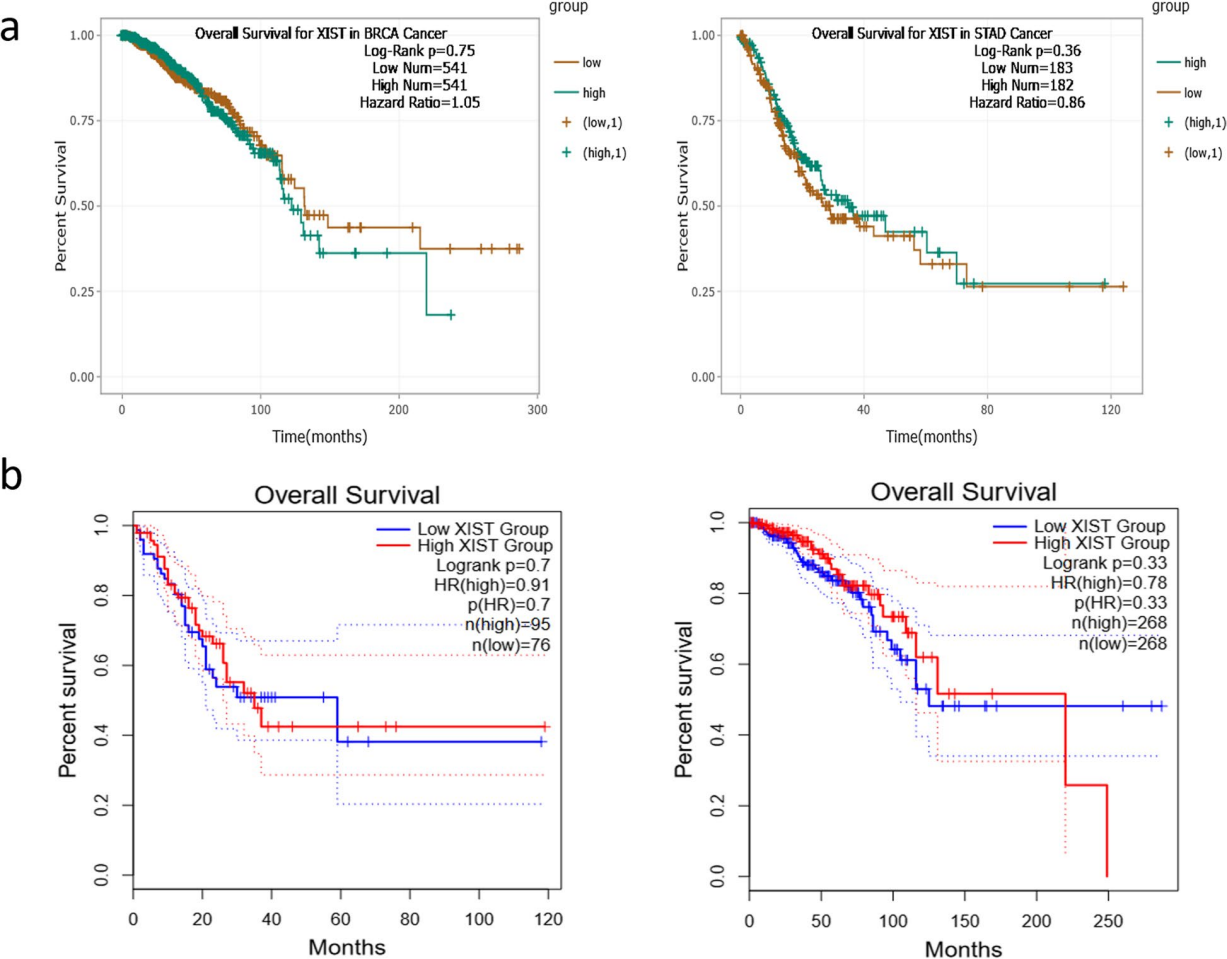
Survival analysis of lncRNA XIST in the BC and GC patients, based on the ENCORI **a** and GEPIA2 **b** online software

**Fig. 16:**
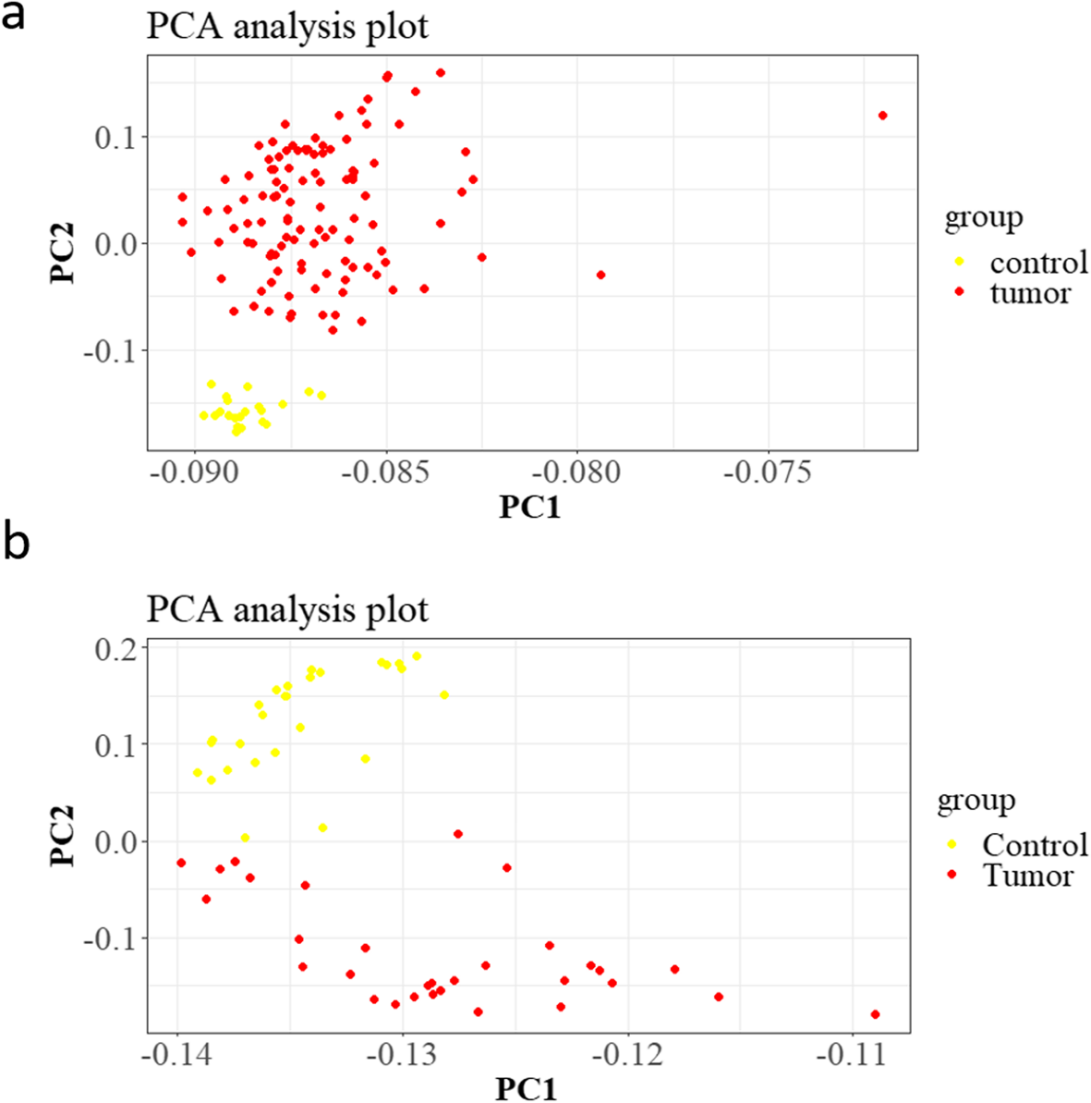
These analyses revealed that these dataset’s control and tumor samples could be separated and are ready for the DEG analysis. Blur color represents the control samples and red color represents the tumor samples

**Fig. 17:**
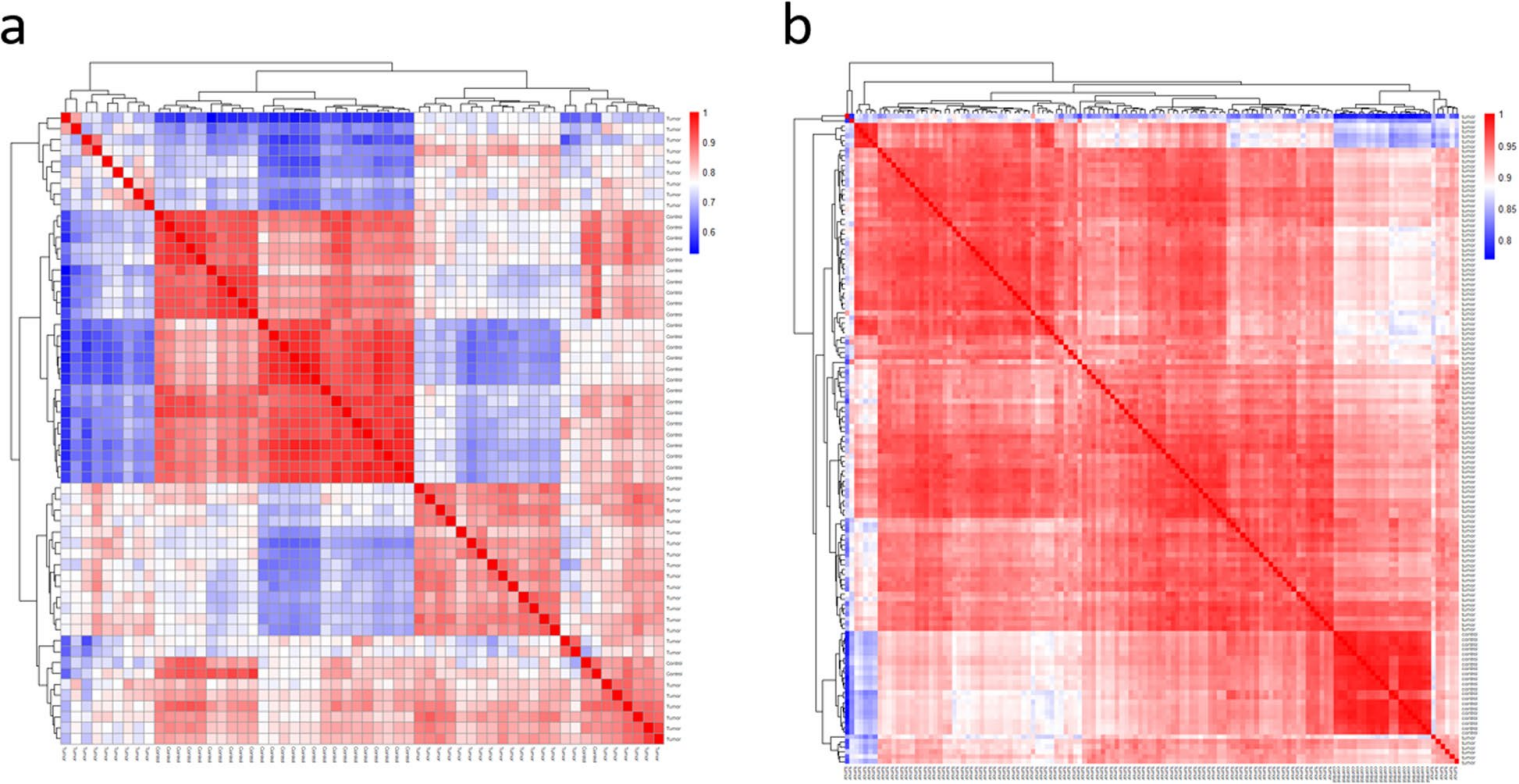
The heatmap of the correlation between microarray samples of BC and GC

**Fig. 18:**
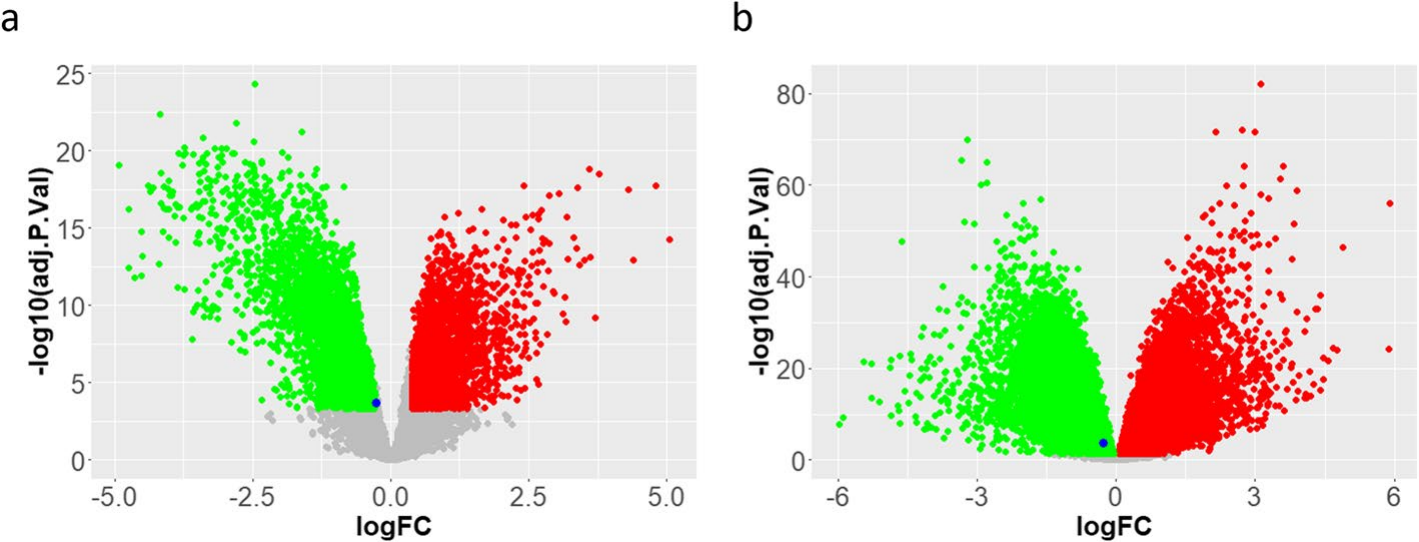
Volcano plot indicates the differentially expressed genes in the BC **a** and GC **b** samples, compared to the control samples. In this plot, red indicates the up-regulated genes, and green indicates the low-expressed genes in these datasets. The lncRNA XIST indicates in these plots by a blue point as a low-expressed gene in the BC and high-expressed genes in the GC samples

Microarray data analysis was performed on BC and GC microarray datasets. The principal component analysis (PCA) and Pearson correlation tests were performed to evaluate the quality of microarray samples (Figs. 15 and 16). DEG analysis revealed that lncRNA XIST has a significant low expression in the BC samples and a high expression in the GC samples (Figs. 17 and 18). Differentially expressed genes in the breast cancer and gastric cancer are provided in the Table 5 and Table 6.

**Table 5:** The list of DEGs in the breast cancer samples, compared to control samples

Gene.Symbol	logFC	*P*.Value	adj.*P*.Val	Down / up
SCARA5	-19.06836248	4.81E-27	51.19456086	Down regulated
ATP1A2	-17.4987381	3.74E-25	46.93195261	Down regulated
SDPR	-16.84157808	2.51E-24	45.06319959	Down regulated
EBF1	-16.77132444	3.08E-24	44.86037459	Down regulated
TNXA	-16.68010511	4.04E-24	44.59613199	Down regulated
CD300LG	-16.39946006	9.28E-24	43.77682645	Down regulated
CA4	-16.3486524	1.08E-23	43.62747145	Down regulated
FIGF	-16.26955502	1.37E-23	43.3943242	Down regulated
ACACB	-16.20488169	1.66E-23	43.20312041	Down regulated
GPC3	-16.13887364	2.03E-23	43.00743765	Down regulated
	-16.12893127	2.09E-23	42.97791647	Down regulated
TNXA	-16.03730336	2.76E-23	42.70527476	Down regulated
SCARA5	-15.98159246	3.26E-23	42.53899498	Down regulated
GHR	-15.7873573	5.90E-23	41.95623203	Down regulated
TNS1	-15.7143933	7.37E-23	41.73609625	Down regulated
BTNL9	-15.63160234	9.51E-23	41.48550021	Down regulated
ACACB	-15.54923357	1.23E-22	41.23532316	Down regulated
LEP	-15.45554168	1.64E-22	40.94970997	Down regulated
GYG2	-15.4506553	1.66E-22	40.9347836	Down regulated
GYG2	-15.3731752	2.11E-22	40.69770045	Down regulated
TOP2A	3.62075011	2.04E-15	8.18E-14	Up regulated
TOP2A	3.202526816	2.70E-15	1.05E-13	Up regulated
RRM2	3.360962774	4.42E-16	2.10E-14	Up regulated
KIAA0101	3.766743769	8.14E-22	3.40E-19	Up regulated
CKS2	3.041350539	3.15E-20	5.73E-18	Up regulated
COL11A1	4.394925107	2.87E-15	1.10E-13	Up regulated
S100P	3.708002723	5.49E-11	6.72E-10	Up regulated
COL10A1	4.312640505	1.48E-20	3.16E-18	Up regulated
RRM2	3.414839829	7.62E-15	2.55E-13	Up regulated
HIST1H2BJ	3.128893785	2.57E-11	3.38E-10	Up regulated
COL10A1	4.801245003	7.24E-21	1.93E-18	Up regulated
NUSAP1	3.184167504	2.36E-18	2.05E-16	Up regulated
GJB2	3.511778652	2.96E-15	1.13E-13	Up regulated
C15orf48	3.154713189	1.77E-12	3.07E-11	Up regulated
INHBA	3.595273877	2.90E-22	1.48E-19	Up regulated
	3.31622272	7.10E-17	4.30E-15	Up regulated
WISP1	3.3945249	1.12E-20	2.47E-18	Up regulated
FOXA1	3.170617626	9.20E-11	1.07E-09	Up regulated
COL11A1	5.051683426	8.98E-17	5.25E-15	Up regulated

**Table 6:** The DEGs in the gastric cancer samples

Gene.Symbol	logFC	*P*.Value	adj.*P*.Val	Down / Up
GKN1	-5.968629214	3.84E-09	9.942157928	Down regulation
GKN2	-5.882742732	1.24E-10	13.31908615	Down regulation
GAST	-5.451885101	1.26E-23	42.98601756	Down regulation
SCGB2A1	-5.275110553	4.36E-23	41.75306449	Down regulation
SST	-5.271075181	2.61E-15	23.95872246	Down regulation
DPCR1	-4.908869154	9.13E-14	20.82228227	Down regulation
KRT20	-4.865974685	3.58E-11	14.53979206	Down regulation
CHGB	-4.657535882	6.42E-25	45.9528731	Down regulation
GIF	-4.657105242	2.44E-09	10.38495073	Down regulation
UPK1B	-4.514057976	3.40E-20	37.0810489	Down regulation
VSIG1	-4.461189551	1.18E-12	20.75471596	Down regulation
RP11-363E7.4	-4.421510772	3.82E-24	44.1766793	Down regulation
SOSTDC1	-4.30898382	2.70E-16	26.20946157	Down regulation
CAPN9	-4.289236342	1.30E-20	41.38701779	Down regulation
AKR1B10	-4.086550468	2.93E-09	10.20824309	Down regulation
MSMB	-4.058405667	2.99E-08	8.025847075	Down regulation
GPR64	-3.92553276	3.19E-23	42.06321501	Down regulation
TFF2	-3.884545478	5.15E-08	7.399901889	Down regulation
AKR1C1	-3.812172981	3.06E-24	44.39661204	Down regulation
ADH1C	-3.810133526	7.61E-12	16.06648431	Down regulation
SFRP2	5.169931068	8.69E-20	1.42E-18	Up regulation
GREM1	4.374570923	1.13E-21	2.35E-20	Up regulation
CYR61	4.209739319	5.04E-31	3.53E-29	Up regulation
CHI3L1	4.17024308	5.91E-17	6.87E-16	Up regulation
PLA2G2A	4.07908169	4.83E-16	4.96E-15	Up regulation
APOD	3.804786642	8.45E-19	1.26E-17	Up regulation
THBS2	3.785907364	4.48E-23	1.18E-21	Up regulation
SFRP4	3.756192618	1.15E-13	8.31E-13	Up regulation
OGN	3.752153645	1.19E-12	7.56E-12	Up regulation
CXCL8	3.658490032	8.44E-11	4.25E-10	Up regulation
RGS1	3.624785789	3.00E-31	2.18E-29	Up regulation
CTHRC1	3.51622428	9.06E-23	2.30E-21	Up regulation
SULF1	3.496280399	4.35E-22	9.85E-21	Up regulation
H19	3.480788458	6.52E-12	3.79E-11	Up regulation
FCGR3A	3.444658873	3.95E-26	1.55E-24	Up regulation
MGP	3.441786496	2.00E-17	2.47E-16	Up regulation
IGFBP4	3.390948421	4.17E-28	2.11E-26	Up regulation
AQP1	3.330599823	1.25E-23	3.42E-22	Up regulation
VMP1	3.288480185	1.28E-50	1.07E-47	Up regulation
FAP	3.284138469	9.55E-23	2.41E-21	Up regulation

## Discussion

To better understand the more accurate function of NEAT1 in the breast cancer and gastric cancer statuses, we designed integrated bioinformatics and experimental expression analyses to find the expression pattern of NEAT1 and related lncRNAs and mRNAs that have RNA interactions with NEAT1. Also, we performed the survival analyses by different methods and software to demonstrate the effects of the changes in the expression in NEAT1 and corresponding mRNA and lncRNA on the survival rate of the patients. Our results indicated that NEAT1 had a significant up-regulation in the samples bigger than 2 cm, compared to the smaller samples. NEAT1 had a significant down-regulation in the gastric cancer samples compared to the control. Also, NEAT1 can be an excellent prognostic biomarker for the Isfahan GC patients based on ROC analysis. The TCGA, ENCORI, and GEPIA2 data analyses and the experimental methods can validate each other in this study.

Furthermore, we demonstrated that NEAT1 had a significant RNA interaction with MTRNR2L8 protein-coding RNA, which had a significant down-regulation in the BC samples. Also, lncRNA XIST had a significant lncRNA-lncRNA interaction with NEAT1. This study demonstrated that XIST had a significant down-regulation in the BC and GC samples. hsa-miR-612 is a critical regulatory microRNA for NEAT1 with more interaction numbers.

Previous studies showed that miR-612 could have a remarkable effect on regulating invadopodia of hepatocellular carcinoma [25]. This microRNA also has a proven role in regulating tumorigenesis in the neurofibromatosis type 1 by involving in the NFKB1-miR-612-FAIM2 signaling pathway [26]. In 2020, Li T et al. showed that the miR-612/HOXA13 signaling pathway could promote cardiomyocyte apoptosis in chronic heart failure [27]. Another significant role of miR-612 that has been proved by Jin Y et al. in 2020 is inhibiting cervical cancer progression. This microRNA can perform this function by targeting NOB1 [28]. Regulation of SEMA4D in the cholangiocarcinoma can be affected by sponging miR-612 via lncRNA LINC01061 [29]. About the role of microRNA-612 in gastric cancer, we find only one experiment, published in 2018 by Liyan Wang et al., that indicated the tumor suppressor effect of microRNA-612 that can be induced by FOXM1 [30]. Also, about the role of miR-612 in the BC, only Hye Kyung Kim et al. revealed that two common single nucleotide polymorphisms (SNPs) within the miR-612 do not affect the breast cancer cell lines [31]. Nevertheless, for the first time, we find a biological interaction between hsa-miR-612 and NEAT1 that can have significant effects on breast cancer and gastric cancer development. Yang X et al. in 2018 revealed that the PTBP3 splicing factor could destroy the splicing balance of NEAT1 and pre-miR-612 [32]. Based on our bioinformatics approach, hsa-miR-612 can regulate the function of NEAT1 by the higher interaction number compared to the other microRNAs, and this mechanism can affect the GC and BC progression.

About the MTRNR2L8, we find no previous research about the role of difference in the expression of MTRNR2L8 in gastric and breast cancer development. So, this is the first investigation about the possible role of MTRNR2L8 in the development of GC and BC. This protein-coding gene can affect BC development by unwanted changes in the expression and can be regulated by NEAT1. Based on the previous investigations, this human peptide is a biological product of the MT-RNR2 gene from mitochondria [33].

About the lncRNA XIST, Yang X et al. showed that the downregulation of lncRNA XIST can lead the CRC cell into proliferation and metastasis [34]. Also, it is revealed that the down-regulation of XIST can inhibit the development of non-small cell lung cancer [35]. This non-coding RNA also can promote the invasion and migration of papillary thyroid cancer cells [36] and pancreatic cancer [37]. About the proven role of XIST in the BC, Xing F et al. at 2018 showed that the loss of XIST can promote the brain metastasis of BC by activating MSN-c-Met and reprogramming the microglia exosomal miRNA [38]. Also, Zheng W et al. showed that the targeting miR-337 by lncRNA XIST can lead the GC cells into migration and proliferation [39]. Also, this lncRNA can promote the progression of GC through TGF-beta 1 signaling pathway by targeting miR-185 [40].

In this experiment, we had some limitations during the study. Some of our plots (including boxplots of GEPIA2 and ENCORI database) are provided by the online database and some of important details (including p-values) may have not good quality. Also, we had limitations in the accessing human clinical samples and the technics for validation of RNA interaction analyses.

**Fig. 19:**
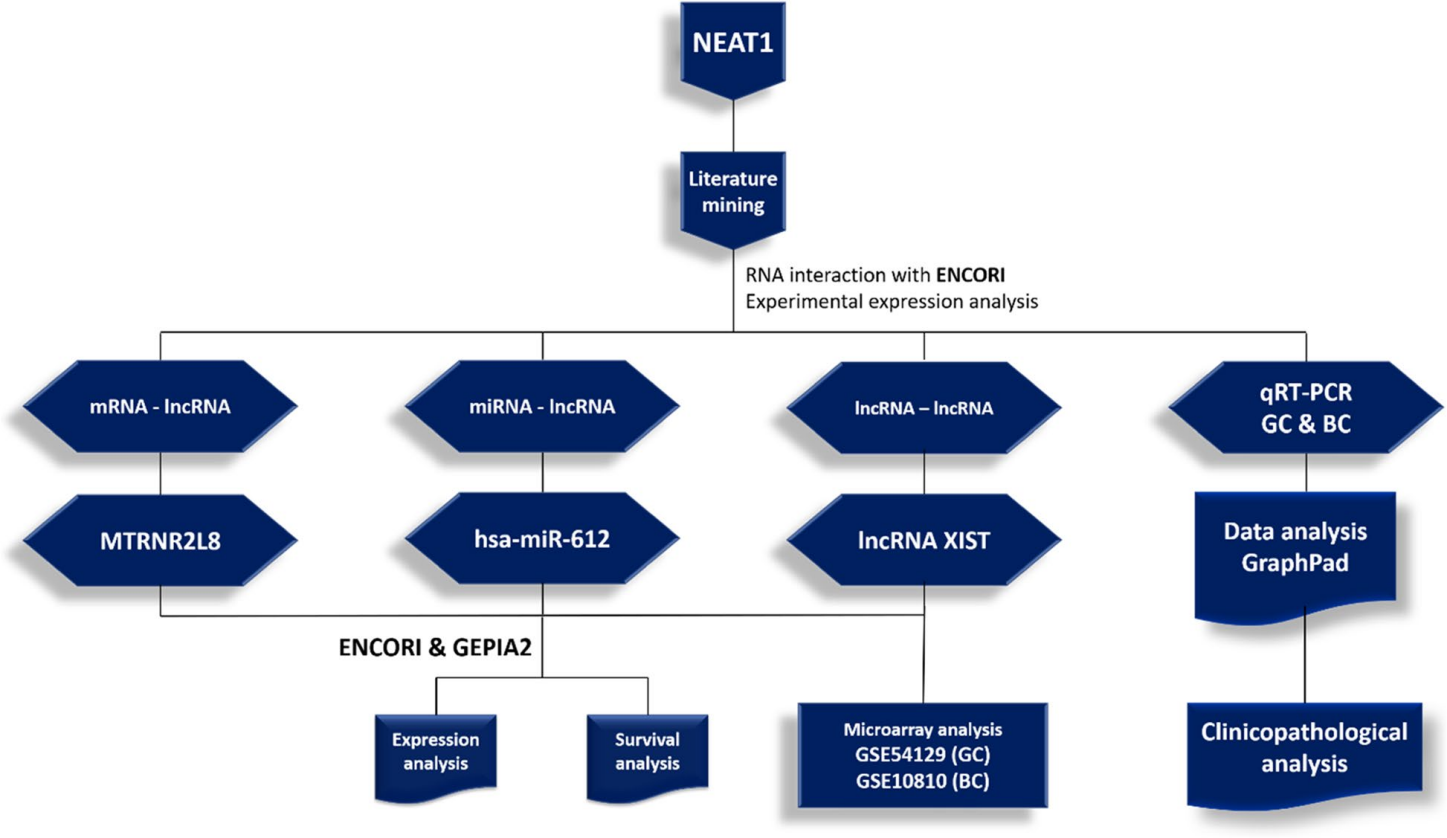
The flowchart of the workflow of this experiment

Based on our investigation and precious studies, the dysregulation of RNA expression and interactions can lead the normal cells into unwanted pathological statuses, including the different cancer types. We aimed to evaluate the expression and interactions of NEAT1 in the BC and GC patients experimentally and bioinformatically. The RIP or luciferase assay method is highly recommended to perform the mentioned interaction analyses. Also, we suggest that the expression level of XIST and MT-RNR2 in the human breast cancer and GC patients to evaluate the accurate expression pattern of these gene and lncRNA in the patients. The flow chart of the workflow in this study is presented in the Fig. 19.

## Conclusion

LncRNA NEAT1 as a significantly dysregulated RNA in the breast cancer and gastric cancer patients have significant interactions with the hub coding (MTRNR2L8) and non-coding RNAs (lncRNA XIST and hsa-miR-612). The changes in the expression level of mentioned RNAs may lead the normal cells of breast and gastric into the tumor status. Furthermore, lncRNA NEAT1 can act as an excellent diagnostic biomarker of gastric cancer patients for distinguishing the tumor and control samples of Isfahan population.

## Data Availability

The datasets used and/or analyzed during the current study are available from the corresponding author on reasonable request.

## Abbreviations

BC: Breast Cancer
GC: Gastric Cancer
NEAT1: Nuclear Enriched Abundant Transcript 1
lncRNA: Long non-coding RNA
FDR: False Discovery Rate
TCGA: The Cancer Genome Atlas
